# Detection of Epstein-Barr Virus in 130 Cases of Eyelid Sebaceous Gland Carcinoma Using In Situ Hybridization

**DOI:** 10.1155/2020/7354275

**Published:** 2020-03-30

**Authors:** Huanhuan Gao, Lijuan Tang, Jianxian Lin, Wenxin Zhang, Yongping Li, Ping Zhang

**Affiliations:** Department of Ocular Pathology, State Key Laboratory of Ophthalmology, Zhongshan Ophthalmic Center, Sun Yat-Sen University, Guangzhou 510060, Guangdong Province, China

## Abstract

**Purpose:**

In this study, we aimed to investigate the presence of Epstein–Barr virus (EBV) in the eyelid sebaceous gland carcinoma (SGC) and its association with the clinicopathologic features.

**Methods:**

One hundred and thirty paraffin-embedded SGC specimens were retrieved from the Clinical Pathology Department of Zhongshan Ophthalmic Center. Epstein–Barr virus-encoded RNA (EBER) was detected with in situ hybridization (ISH) using the Leica BOND system autostainer. The age and gender distributions of all patients were analyzed and compared with earlier reports. Pearson's *χ*^2^ and Fisher's exact tests were used to determine the association between clinicopathological features such as age, gender, laterality eye, tumor basal dimension, degree of tumor differentiation, and EBER positivity. Likewise, the relationship between the grade and tumor basal dimension in EBER-positive SGC of the eyelid was analyzed.

**Results:**

Thirty-four out of one hundred and thirty (26.2%) eyelid SGC specimens were positively stained for EBER. The age range of highest incidence was 46–75 years, and the female to male ratio was 1 : 0.9. No significant correlation was found between EBER-positivity and age (*p* = 0.5370), gender (*p* = 0.4758), and degree of tumor differentiation (*p* = 0.7787). However, EBV positivity was strongly correlated with the right eye (*p* = 0.0287), the tumor basal dimension (*p* = 0.0001). EBV positivity grade presented statistically associated with tumor size (*p* = 0.0329).

**Conclusion:**

We conclude that ISH is a sensitive method to identify EBV in SGC of the eyelid. A possible causal association of EBV in SGC patients is suggested by high frequency of EBER-ISH positivity and its association with the clinicopathologic features.

## 1. Introduction

Sebaceous gland carcinoma (SGC) of the eyelid is the second most common malignant tumor among all eyelid malignancies after basal cell carcinoma (BCC). It arises from the eyelashes (i.e., glands of Zeis) or the tarsal plate (i.e., meibomian glands) and usually occurs in females older than 50 years of age [1, 2]. Previous studies have defined the risk factors for SGC of the eyelid to be pathogenically related and to include exposure to irradiation, immunosuppression, and the use of diuretics [3]. A study aiming to identify additional risk factors has reported a possible viral etiology. In Japan, for instance, HPV infections exist in a high percentage of individuals with SGC of the eyelid [4]. Despite this, virus infection has not been much considered for SGC of the eyelid.

Epstein–Barr virus (EBV) is a ubiquitous DNA virus of the herpes family that infects 90% of humans [5]. Numerous studies have reported a strong correlation between EBV infection and gastric cancer [6], lung cancer [7], breast cancer [8], lymphoma [9], and other tumors [10–12]. However, there is no study on the relationship between the EBV and SGC of the eyelid.

The purpose of this study was to investigate the correlation between EBV infection and SGC of the eyelid. Using EBV-encoded RNA (EBER) in situ hybridization (ISH), we screened for the presence of the EBV in SGC of the eyelid and determined if a relationship exists between EBV infection and SGC tumorigenesis. To our knowledge, this is the first study to use ISH to investigate the correlation between EBV infection and SGC of the eyelid. Our results may deepen our understanding of the etiology of SGC of the eyelid of patients and provide a reference for treatment of this disease.

## 2. Methods

### 2.1. Patients and Samples

Formalin-fixed, paraffin-embedded specimens from 130 patients diagnosed as SGC of the eyelid during a six-year period from January 2012 to January 2018 were retrieved from the Clinical Pathology Department of Zhongshan Ophthalmic Center, Sun Yat-sen University. Cases with nonocular adnexal were excluded from the study and with available paraffin blocks of the tissue were studied. The clinical and pathological data were abstracted from clinical records. This study was approved by the Medical Ethics Review Board of Zhongshan Ophthalmic Center, Sun Yat-sen University.

### 2.2. Detection of EBER

**Figure 1:**
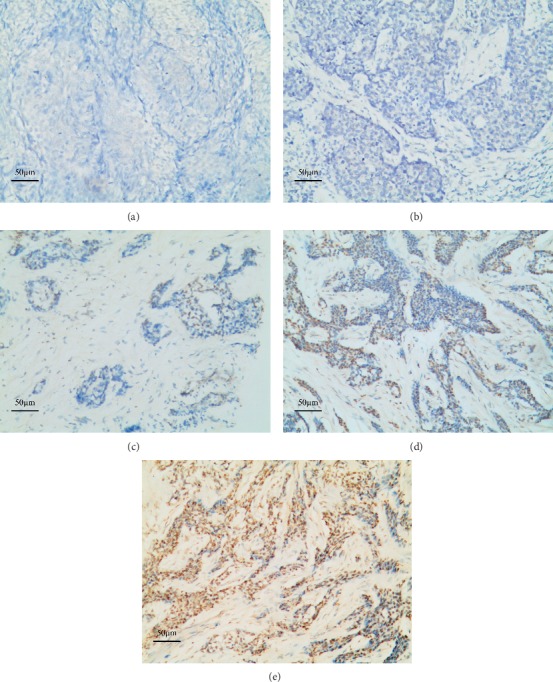
Grading of the EBER reaction (ISH) in sebaceous carcinoma of the eyelid. Sebaceous carcinoma of the eyelid, magnification 100x. Negative control (a), Grade 1+ (b), Grade 2+ (c), Grade 3+ (d), and Grade 4+ (e).

Four micron-thick tissue sections were collected on electrostatic-charged slides and dried for 30 min at 60°C. The slides were covered by Bond Universal Covertiles (Leica Microsystems) and placed into the Bond-Max Autostainer (Leica Biosystems, Melbourne, Australia). Through use of the Leica BOND-MAX system (Leica Biosystems, Melbourne, Australia), slides were automatically deparaffinized with BondDewax solution 3 times at 72°C for 1 minute and then rinsed 3 times with alcohol and 4 times with BondWash solution. After 15 minutes of incubation with enzyme 1 (Bond Enzyme Pretreatment Kit, CAT# AR9551) at 37°C, fluorescein-conjugated probe (Bond Ready-to-Use ISH EBER Probe, CAT# PB0589) was placed on the slides and incubated for 2 hours at 37°C. After a peroxide block for 5 minutes, slides were incubated with antifluorescein antibody for 15 minutes, post primary reagent for 8 minutes, and polymer for 8 minutes, all at room temperature. All the previous steps were followed by 4 rinses with BondWash solution. The final polymer incubation was followed by 2 BondWash and 1 distilled water rinses. Staining was performed with Mixed DAB Refine for 10 minutes at room temperature, followed by 3 distilled water rinses, a 5-minute hematoxylin counterstain, and 1 rinse in BondWash and then 1 distilled water rinse, dehydration, clearing, and cover slipping. EBER-positive nasopharyngeal carcinoma served as the positive control, whereas the normal tissue served as the negative control. The positive cells by the appearance of a dark brown precipitate were counted under a microscope at a high magnification. This evaluation was performed by two pathologists (Zhang, Tang) and scored according to the percentage of positive cells (Figure 1). The images were photographed using a digital camera attached to an Olympus BX1 light microscope (Tokyo, Japan). Referring to the previous article [13], the criteria used in scoring and grading the intensity of the EBER staining is as follows.

  0, no reaction in all cells (Figure 1(a))
  1+, scarce EBER-positive cells (<5%), suggestive of isolated reactive or activated normal cells and/or immunoblasts (Figure 1(b))
  2+, few EBER-positive neoplastic cells (5–25%) (Figure 1(c))
  3+, some neoplastic cells (26–75%) EBER positive (Figure 1(d))
  4+, most neoplastic cells (>75%) EBER positive (Figure 1(e))

Grades 2+, 3+, and 4+, with a cutoff value of >5% EBER-positive neoplastic cells, were considered EBV positive, and grade 1+, with inadequate or equivocal positive neoplastic cells (a cut-off value <5%), were excluded from this study.

### 2.3. Statistical Analysis

Pearson's χ^2^ and Fisher's exact tests were used to determine the association between positive EBER staining with the age, gender, laterality eye, tumor basal dimension, or degree of tumor differentiation, respectively. Correlation of the grade with tumor basal dimension in EBER-positive SGC of the eyelid was also performed using Fisher's exact test. A *p*-value <0.05 was indicated statistically significant.

## 3. Results

### 3.1. Analysis of Age and Gender Data

A total of 130 patients with SGC of the eyelid were divided into different age groups. The age and gender distributions are presented in Table 1. The age of patients ranged from 28 to 81 years, with a mean age of 57.9 years. With 62 males and 68 females, there was a slight preponderance of females to males as the male to female ratio was 0.9 : 1. There was a peak from 46 to 75 years of age, coinciding with the high-risk incidence groups (i.e., 46–55, 56–65, and 66–75) (Table 1).

### 3.2. EBER Positivity Analysis

There were 34 out of 130 patients (26.2%) with EBER-positive SGC of the eyelid. Overall, there were five cases (14.7%) graded as 2+, 12 (35.3%) cases graded as 3+, and 17 (50%) cases graded as 4+. These results are presented in Table 2.

### 3.3. Relationship between EBV Reactivity and Clinicopathological Features

The correlation between EBV reactivity and clinicopathological features is summarized in Table 3. There were 24 out of 34 patients (70.6%) with EBER-positive SGC of the eyelid in the high-risk incidence group (age range, 46–75 years). The remaining six cases were 45 years of age or younger, and four cases were older than 76 years of age. Furthermore, there were 18 male and 16 female patients with EBER-positive SGC of the eyelid. Twenty-three cases (67.6%) were the right eye, whereas the rest (32.4%) were the left eye. A statistical significant difference with *p* = 0.0287 was found between the laterality eye and EBV positivity. The tumor basal dimension ranged from 1 to 50 mm, and majority of EBER-positive cases (70.6%) had a tumor size more than 10 mm as shown in Table 3. The remaining patients were EBER-negative. The degree of tumor differentiation was summarized as number and percent and compared to the EBV-positive rate. As a result, there was not any statistically significant difference between EBV positivity and age (*p* = 0.5370), gender (*p* = 0.4758), and degree of tumor differentiation (*p* = 0.7787). However, 24 out of 34 patients had a tumor size more than 10 mm, indicating that EBV positivity was strongly correlated with the tumor size (*p* = 0.0001).

### 3.4. Relationship of the Grade and Tumor Basal Dimension in EBER-Positive SGC of the Eyelid

Regarding the association between the EBV positivity grade and tumor size, there was a statistical significant difference between EBV presence grade and tumor size (*p* = 0.0329) (Table 4).

## 4. Discussion

Eyelid SGC is slow-growing but a highly invasive and malignant cancer of the eyelid [14]. This neoplasm always masquerades itself as chalazion, chronic conjunctivitis, or other tumors, resulting in delays in diagnosis and subsequent morbidity and mortality [15, 16]. Studies have shown that EBV infection may be critical in the development of malignant tumors [17–19]. To date, this is the first study to use ISH to investigate the correlation between EBV infection and SGC of the eyelid.

In this study, the age of patients ranged from 28 to 81 years, with a mean age of 57.9 years. There were 21 out of 130 patients (16.1%) 45 years of age or younger (Table 3). As previously reported [3], SGC generally occurs in older individuals, with those aged 46–75 years being in the high-incidence group. These results are consistent with those of another study that reported SGC of the eyelid arising in the sixth or seventh decade of life (57–72 years) [2]. However, SGC can also occur in older children and young adults [20]. On the other hand, females (52.3%) were more likely to be affected by the disease than males (the female : male ratio was 1 : 0.9), which is in agreement with another study that showed a female preponderance for SGC, with the female : male ratio at 1.4 : 1 [2]. In reality, though, there was no correlation between positive EBER staining and age and gender after Pearson's *χ*^2^ and Fisher's exact tests were carried out.

EBV-positive in our study presented a significant difference with the right eye (*p* = 0.0287). Rosenbach [21] claimed that most people had a dominant eye, even though each of their two eyes in isolation may provide equal vision and in unequal vision, the dominant eye is not always the eye with better visual acuity. Hillemans [21] found right ocular dominance in 40% of patients, left ocular dominance in 20% of patients, and uncertain results in 40% of patients. Their interpretation of this finding is that the right eye, apparent as ocular dominance, is more susceptible to the EBV latency condition and presumably due to the degree of photodamage (and associated DNA damage) by the decrease of the host's immunity and stimulation of condition and so on. Meanwhile, EBER positivity was strongly correlated with tumor basal dimension, wherein EBER positivity was found to be significantly more in tumors ≥10 cm (70.6%) as compared to tumors <5 mm (0%) and tumors in 6–10 mm (29.4%). These observations are concordant with the research [22] about the EBV association with prognostic parameters and have found EBV positivity to be associated with aggressive features like larger tumor size, while the degree of tumor differentiation data did not show a strong association with EBV positivity (*p* = 0.7787). Furthermore, studies [23–26] have reported several factors including larger lesions and histopathologic features, such as different ethnicities, poor differentiation, multicentric origin, pagetoid spread, and molecular marker expression determine the prognostic factors for local recurrence, metastasis, and survival in patients with SGC of the eyelid. Concerning these, their association with EBV needs to be further studied.

There were 34 out of 130 patients (26.2%) with EBER-positive SGC of the eyelid (Table 2). There were 17 cases graded as 4+, representing >75% EBER-positive neoplastic cells. Other ISH studies have reported EBER reactivity in 22.6% (14/62) of nodal or extranodal T- and NK- cell lymphomas cases [13], with the highest reactivity (30.1%) being in breast tumors [27] and the lowest reactivity (10%) being in gastric tumors (10%) [28]. Considering the strong EBER reactivity that was restricted to the nuclei of tumors, we presume that there is a close relationship between EBV reactivity and SGC of the eyelid. This finding has potential preventive and therapeutic relevance, which might have a significant impact on the patient management. For example, the potential benefit of antivirals and intravenous immunoglobulin has been studied for the prevention of PTLD in EBV-seronegative patients who are receiving transplants from EBV-seropositive donors [29].

The correlation between EBV reactivity and malignant tumors has been investigated extensively using varied techniques such as the polymerase chain reaction (PCR), laser capture microdissection (LCM), immunohistochemistry (IHC), southern blot hybridization, and ISH [30]. These methodologies, however, associate with several shortcomings. For instance, PCR is highly sensitivity, and it can detect expression EBV genes but it cannot detect the viral genome in B-cells and cannot differentiate EBV in tumor cells from EBV in surrounding lymphocytes. LCM is always used to separate malignant cells from surrounding lymphocytes before PCR testing. IHC is one of the extensively used and easily amenable techniques for EBV detection, and it permits direct visualization of the viral proteins within tumor cells; however, the cross reactivity of the antibodies questions its specificity. Southern blot hybridization analysis is less sensitive than PCR for detecting viral DNA. A previous study has even suggested that ISH is still used to confirm the PCR positive cases [31]. With regards to sensitivity and specificity, ISH is the gold standard in the detection of EBV infection [30, 31]. Moreover, ISH technique for EBER has not been reported in specimens of SGC of the eyelid.

EBER expression is detected in most of the EBV-positive carcinoma patients. However, the relevance between EBER expression and the clinical outcome has been rarely reported in ophthalmic tumors. This paper aims to assess the possible correlations of EBER expression and clinical parameters and its potential prognostic predictive ability in SGC patients' outcomes. In our study, clinical information of 130 patients with SGC was included into analysis, such as age, gender, laterality eye, tumor basal dimension, histological differentiation, and with EBER positivity. However, due to the lack of follow-up data, survivals were not collected, so the association between EBER-positive and EBER-negative cases in the patient prognosis levels was not compared. The etiologic importance of EBV in the pathogenesis of SGC is yet to be fully elucidated and worth further studies.

Above all, to the best of our knowledge, this is the first study to investigate the relationship between EBV infection and SGC of the eyelid using EBER-ISH technique. Further studies are allowed to elucidate the EBV as a risk factor for bad prognosis, with the ultimate goal of having therapeutic significance as a potential target.

## Figures and Tables

**Table 1:** Age and gender distribution of patients with SGC of the eyelid.

Age (in years)	Gender (no. of patients)		Total
	Male	Female	
28–35	4 (6.5)	3 (4.4)	7 (5.4)
36–45	4 (6.5)	10 (14.7)	14 (10.8)
46–55	15 (24.2)	17 (25)	32 (24.6)
56–65	16 (25.8)	20 (29.4)	36 (27.7)
66–75	18 (29.0)	13 (19.1)	31 (23.8)
76–81	5 (8.1)	5 (7.4)	10 (7.7)
Total (%)	62 (47.7)	68 (52.3)	130 (100)

**Table 2:** Grade distribution of patients with EBER-positive SGC of the eyelid.

Grade	EBER (+)
	No. of cases
2+	5 (14.7)
3+	12 (35.3)
4+	17 (50)
Total	34

**Table 3:** Association of EBV reactivity with clinicopathological features.

Parameter	Patient no.	EBER (+)	EBER (−)	*p*-value
	(n = 130)	(*n* = 34)		
Age (years)				
≦45	21 (16.1)	6 (17.6)	15 (15.6)	0.5370
46–75	99 (76.2)	24 (70.6)	75 (78.1)	
>76	10 (7.7)	4 (11.8)	6 (6.3)	

Gender				
Male	62 (47.7)	18 (52.9)	44 (45.8)	0.4758
Female	68 (52.3)	16 (47.1)	52 (54.2)	

Laterality				
Right	67 (51.5)	23 (67.6)	44 (45.8)	0.0287
Left	63 (48.5)	11 (32.4)	52 (54.2)	

Tumor basal dimension (mm)				
≦5	9 (6.9)	0	9 (9.4)	0.0001
6–10	70 (53.8)	10 (29.4)	60 (62.5)	
>10	51 (39.2)	24 (70.6)	27 (28.1)	

Differentiated degree				
Well	19 (14.6)	4 (11.8)	15 (15.6)	0.7787
Moderately	48 (36.9)	14 (41.2)	34 (35.4)	
Poorly	63 (48.5)	16 (47.1)	47 (49)	

**Table 4:** The relationship between the grade and tumor basal dimension in EBER-positive SGC of the eyelid. T1 was the tumor basal dimension between 6 and 10 mm. T2 was the tumor basal dimension more than 10 mm.

EBER-positive grade	EBER-positive tumor size		*p*-value
	T1	T2	
2+	4 (40)	1 (4.2)	0.0329
3+	2 (20)	10 (41.7)	
4+	4 (40)	13 (54.2)	
Total	10	24	

## Data Availability

The data used to support the findings of this study are restricted by Medical Ethics Review Board of Zhongshan Ophthalmic Center in order to protect patient privacy. Data are available at the State Key Laboratory of Ophthalmology for researchers who meet the criteria for access to confidential data.

## Supplementary Materials

Supplementary Materials Figure 1: sebaceous carcinoma of the eyelid, HE, magnification 100x. Figure 2: sebaceous carcinoma of the eyelid, HE, magnification 400x.
