# Supplementary figures and images for: Detection of Epstein-Barr Virus in 130 Cases of Eyelid Sebaceous Gland Carcinoma Using In Situ Hybridization
Source: J Ophthalmol. 2020 Mar 30;2020:7354275. doi: 10.1155/2020/7354275 (PMC7152957; doi:10.1155/2020/7354275)

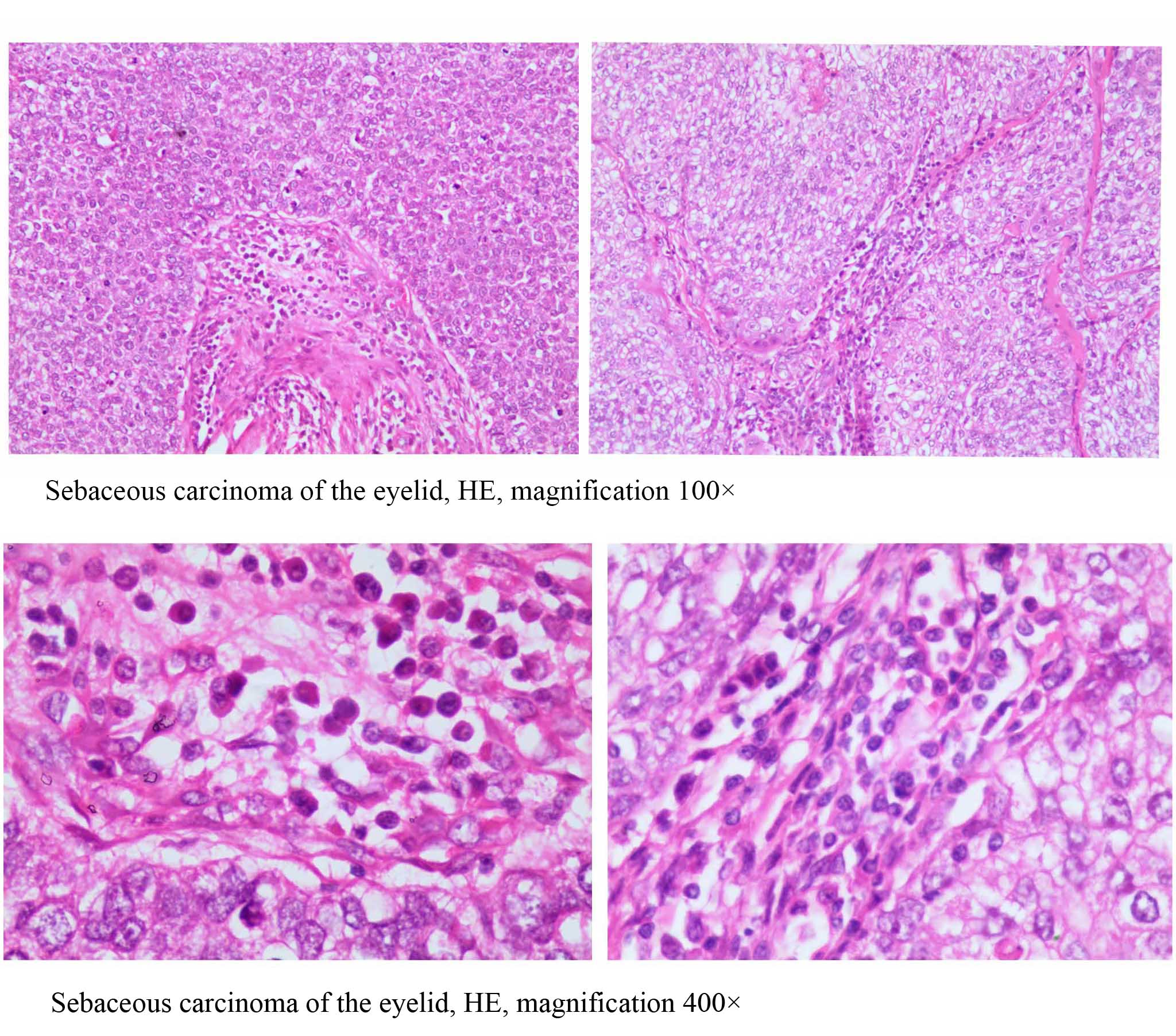

Supplement: Supplementary Materials — Figure 1: sebaceous carcinoma of the eyelid, HE, magnification 100x. Figure 2: sebaceous carcinoma of the eyelid, HE, magnification 400x. [file 7354275.f1.jpg]
